# Grasping Weber’s Law in a Virtual Environment: The Effect of Haptic Feedback

**DOI:** 10.3389/fpsyg.2020.573352

**Published:** 2020-11-19

**Authors:** Aviad Ozana, Sigal Berman, Tzvi Ganel

**Affiliations:** ^1^Department of Psychology, Ben-Gurion University of the Negev, Beer-Sheva, Israel; ^2^Zlotowski Center, Ben-Gurion University of the Negev, Beer-Sheva, Israel; ^3^Department of Industrial Engineering and Management, Ben-Gurion University of the Negev, Beer-Sheva, Israel

**Keywords:** perception and action, grasping, Weber’s law, 2D objects, virtual environment, object perception

## Abstract

Recent findings suggest that the functional separation between vision-for-action and vision-for-perception does not generalize to situations in which virtual objects are used as targets. For instance, unlike actions toward real objects that violate Weber’s law, a basic law of visual perception, actions toward virtual objects presented on flat-screens, or in remote virtual environments, obey to Weber’s law. These results suggest that actions in virtual environments are performed in an inefficient manner and are subjected to perceptual effects. It is unclear, however, whether this inefficiency reflects extensive variation in the way in which visual information is processed in virtual environments or more local aspects related to the settings of the virtual environment. In the current study, we focused on grasping performance in a state-of-the-art virtual reality system that provides an accurate representation of the 3D space. Within this environment, we tested the effect of haptic feedback on grasping trajectories. Participants were asked to perform bimanual grasping movements toward the edges of virtual targets. In the haptic feedback condition, physical stimuli of matching dimensions were embedded in the virtual environment. Haptic feedback was not provided in the no-feedback condition. The results showed that grasping trajectories in the feedback, but not in the no-feedback condition, could be performed more efficiently, and evade the influence of Weber’s law. These findings are discussed in relevance to previous literature on 2D and 3D grasping.

## Introduction

People interact with physical objects in their surroundings by reach-to-grasp movements. Current advances in immersive technology aim to simulate a similar sense of control when interacting with virtual objects within virtual environments. Recent studies, however, suggest that virtual interactions are (still) performed differently from interactions with 3D objects in the physical environment (Holmes and Heath, 2013; Freud and Ganel, 2015; Ozana and Ganel, 2018, 2019a). For instance, grasping gestures toward physical 3D objects are typically performed analytically. In particular, the shaping of the grip aperture is unaffected by tasks-irrelevant, perceptually driven information about objects and their surroundings (Aglioti et al., 1995; Ganel and Goodale, 2003; Ganel et al., 2008; Chen et al., 2015; Namdar et al., 2018 but see Franz and Gegenfurtner, 2008; Kopiske et al., 2016). These findings have been attributed to the proposed functional separation between visual perception and visual control of action (Goodale and Milner, 1992; Milner and Goodale, 2008; but see Glover and Dixon, 2001; Smeets and Brenner, 2008; Rossit et al., 2018 for different views). These findings have been recently extended to two-hand, bimanual motor control (Ganel et al., 2017; Ozana and Ganel, 2020). Yet, unlike grasping movements toward physical objects, previous evidence shows that when 2D objects are used as targets, actions toward these objects become less efficient and are performed in a relative rather than analytic manner. Such actions are susceptible to perceptual heuristics (Holmes and Heath, 2013; Freud and Ganel, 2015; Ozana and Ganel, 2018, 2019a; Ganel et al., 2019). This evidence suggests that efficient visuomotor control is compromised when 2D objects are used as targets.

Compelling evidence for the difference between visuomotor interactions with 2D and 3D objects comes from experiments that looked at the adherence of grasping movements to Weber’s law. According to Weber’s law, the smallest detectable change along the size of an object is proportional to its initial size. The Just noticeable differences (JNDs), therefore, linearly increases with size, an indication of the relative nature of human perceptual resolution. Previous studies have shown that for grasping movements performed toward physical objects, JNDs (measured at the point in which the maximum grip aperture, MGA, is achieved) do not increase with the target’s size, in violation of Weber’s law (Ganel et al., 2008, 2014; Heath et al., 2011; Ganel, 2015). However, when grasping movements are directed to 2D targets, grasping apertures show an abnormal pattern of adherence to Weber’s law (Holmes and Heath, 2013; Hosang et al., 2016; Ozana and Ganel, 2019a; Ozana et al., 2020). These findings, again, suggest that visuomotor interactions with virtual objects are subjected to perceptual and relative heuristics.

Physical objects provide rich visual cues about surface, depth, and perspective, and provide haptic feedback upon touch. Such objects may afford a sense of agency upon the interaction, a sense of agency that may be compromised in virtual interactions (Freud et al., 2018; Ozana and Ganel, 2019a). It is unclear, however, which of the visual and non-visual characteristics that lack in virtual interactions contribute to the observed difference between grasping trajectories toward 3D and 2D objects. For example, 2D grasping does not provide object-specific haptic information upon touch. This feedback may be used to calibrate and to refine visuomotor interactions in repeated trials (Davarpanah Jazi and Heath, 2016; Hosang et al., 2016; also see, Bingham et al., 2007; Johansson and Flanagan, 2009; Whitwell et al., 2016; Cesanek and Domini, 2017; Kopiske et al., 2017). The results of a recent study from our lab, however, showed that the provision of haptic feedback did not change the nature of the grasping trajectories in virtual settings (Ozana et al., 2018). In this study, participants were asked to “grasp” virtual objects within a remote virtual environment, with the use of a haptic telerobotic system that provided digitized representation of the location of the fingers, as well as object-specific haptic feedback upon virtual interception of the object. Although the system we used could potentially emulate visuomotor interactions with objects within the computerized space, the results showed that grasping trajectories within this system were atypical; Just as in the case of interactions with 2D images of objects, grip apertures obeyed to Weber’s law. Furthermore, the pattern of adherence to Weber’s law in the haptic feedback condition was similar to that obtained in a matched no-feedback condition. These findings converge with previous results (Afgin et al., 2017), to suggest that visuomotor control in virtual environments relies on less efficient, relative computations of size. Such inefficiency might be accounted for by an inadequate level of authenticity of the virtual system in terms of the quality of the visual and tactile feedback it provides (Ozana et al., 2018). It is possible that unreliable haptic feedback may not evoke the dedicated set of computations that support normal visuomotor control during interactions with physical objects. In the current study, we used an advanced VR system to simulate a more reliable sense of control of virtual objects. We tested whether such interactions could be supported by efficient visuomotor control that evades the influence of Weber’s law. To achieve this purpose, we tested the potential contribution of informative haptic feedback upon touch.

VR systems are considered as hallmarks of immersive technology. Modern devices are capable of providing rich 3D binocular and monocular cues, as well as motion parallax cues, which correspond to the observer’s position with respect to virtual objects in the digitized space. Compared to older virtual settings, modern virtual environments are designed to simulate a reliable sense of control in interactions with virtual objects and to allow natural and efficient visuomotor control within the virtual settings. Nevertheless, state-of-the-art VR systems still suffer from technical drawbacks in precision and temporal synchronization between their various components. For example, current technology does not provide complete transparency between the operator’s movement and its digitized representation (Furmanek et al., 2019). As noted above, these technical disadvantages might compromise the sense of agency or potential interaction with the target. Hence, it is still unclear whether virtual interactions using current VR technology could operate in an efficient manner.

We note, that inefficient visuomotor control in virtual interactions could also be attributed to lack of familiarity with the task within the virtual settings. In the context of visual illusions, for example, it has been demonstrated that unpracticed, awkward grasping movements are more likely to be prone to illusory effects, compared to highly practiced precision grasps, In addition, the lack of efficiency during awkward grasping can be attenuated after extensive training (Gonzalez et al., 2008). In the current study, besides from studying the effect of haptic feedback on motor control in the VR, we were also able to look at the effect of practice, by comparing performance during sequential experimental blocks (bins) throughout the task.

Therefore, the present investigation was aimed at examining whether the typical pattern of grasping trajectories that characterizes 3D grasping would extend to actions toward virtual targets in a virtual space. To this end, participants performed bimanual grasping movements within a state-of-the-art VR environment that permits interactions with large objects using two-handed grasping. We note that while most of the cited work here focused on unimanual, precision grasps, two recent studies from our lab demonstrated action-perception dissociations during bimanual grasping (Ganel et al., 2017; Ozana and Ganel, 2020). Relevant to the current study, a dissociable pattern of adherence to Weber’s law was recently found between bimanual grasping and perceptual adjustments (with the former type of response violating Weber’s law) (Ganel et al., 2017). As in previous studies, the current study utilizes the adherence to the psychophysical principle of Weber’s law as a tool to probe the nature of the underlying processes (Ganel, 2015; Ganel et al., 2017). To test the potential contribution of haptic feedback to the effect, we manipulated the availability of haptic information at the end of the grasp. In Experiment 1, different-sized virtual targets were presented for grasp while haptic feedback was not provided upon touch. In Experiment 2, we used the same experimental settings, but now haptic feedback was provided upon touch from a matching set of physical objects (see Hosang et al., 2016). Could grasping in a 3D VR environment escape the influence of Weber’s law? What contribution does haptic feedback have to grasping performance within VR?

## Experiment 1

### Materials and Methods

#### Participants

Fourteen healthy undergraduate students (six males, average age= 25.6, *SD* =1.3) participated in the experiment for the equivalent of 15$. All of the participants provided informed consent, which was approved by the BGU ethics committee.

#### Apparatus and Stimuli

Participants sat on a height-adjustable chair. An HTC Vive system that includes a head-mounted display (AMOLED, 1,080 × 1,200 pixels per eye, 90 Hz) and two motion trackers (SteamVR tracking Inc.) was used to display the virtual environment, and to capture movement within the environment. The apparatus tracked the 3D position of two controllers attached separately to the participant’s left and right forearms (90 Hz sampling rate). A TouchDesigner software (version 2018.27300, Derivative, Toronto, ON) was used to control trial sequence and stimulus presentation.

**FIGURE 1:**
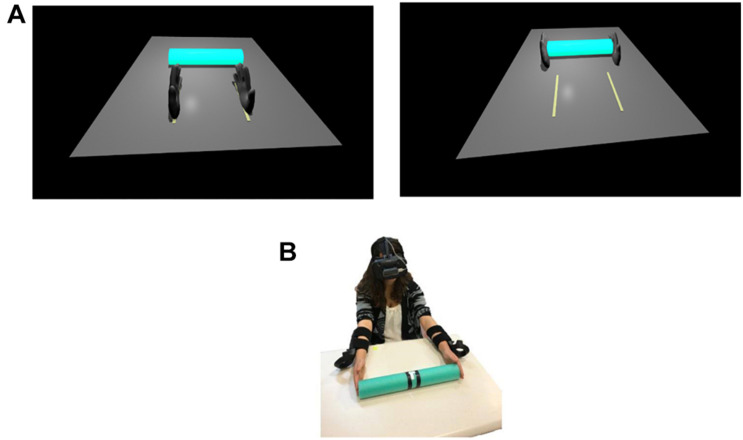
Stimuli and experimental design. Participants performed bimanual grasping movements toward virtual objects presented in the virtual environment. Illustration of the participant view of the virtual environment **(A)**. Illustration of the experimental procedure in Experiment 2 (feedback condition) **(B)**.

Four tube-shaped virtual objects of different sizes (programmed to appear as 15, 25, 35, and 45 cm in length, 4 cm in height, and 5 cm in depth) were used as targets. In each trial, one target object was presented in the center of the 3D virtual environment that was constructed based on the physical environment of the lab. Two virtual hands represented the location of the motion trackers, which were attached to the participant’s forearms, within the virtual environment (Figure 1).

In a recent study, we found that actions toward virtual targets are prone to speed-precision tradeoff effects (Ozana and Ganel, 2019b), an increase in the aperture velocities with target size that can lead to a pattern of scalar variability (a linear increase of the within-subject SDs of the response, as predicted by Weber’s law) during grasping (Foster and Franz, 2013; Ganel et al., 2014, 2017; Ozana and Ganel, 2019b). To avoid the potential confounding effect of aperture velocities on the pattern of adherence to Weber’s law, the initial distance between the participant’s hands was dynamically adjusted to each target size. This procedure has been shown effective in previous research to attenuate the relation between velocity and size, accounting for the possibility of speed-precision tradeoff effects (Ganel et al., 2014, 2017; Ozana and Ganel, 2019b). Notably, adjusting the initial distance between the hands can also reduce the inherent relation between movement time and target size, which can lead to spurious grip scaling in normalized movement procedures (such as the one used here) (Whitwell and Goodale, 2013). Two virtual strips in four different distances were used as starting positions. The distance between the lines was always 5 cm smaller than the target object’s length (10, 20, 30, 40 cm). Velcro strips (4 cm in length) were used to provide haptic feedback for each starting point. The distance between the starting position and the target object was 25 cm (measured from the center of the Velcro strip to the target edges). The participant’s viewing distance from the target object was about 35 cm.

#### Procedure

Prior to each trial, the participants placed their right and left hands on the virtual strips (starting position). The participants were asked to “grasp” the edges of the target upon hearing a “go” signal, and to keep their hands on the stimulus edges until a second tone was presented. No haptic feedback was provided in Experiment 1. Once the second tone was presented, participants returned their hands to the starting position and waited for the beginning of the next trial. The go signal was presented 1 sec after the visual presentation of the target, and the second tone was presented 4 sec after the first one.

After a short equipment calibration, during which the participants were acquainted with the virtual environment, each participant underwent three sequential experimental blocks, in which each stimulus was presented 15 times in a pseudo-randomized order (180 trails in total).

#### Data Analysis

The 3D trajectories of the hands were recorded for each trial and were analyzed offline using MATLAB software (Version 9.0, The Mathworks, Natick, MA). The grip aperture was computed as the Euclidean distance between the two trackers (after the positions of both markers were transformed into a common coordinate system using homogeneous transformations). The aperture data were filtered offline using a standard 2-way (zero lag), low-pass, third-order Butterworth filter with a 6 Hz cutoff. The cutoff frequency was verified visually with the data. Grip aperture tangential velocity was computed by differentiating the vector of the grip aperture. Movement onset was set at the point in time after the presentation of the go command, in which the grip aperture’s tangential velocity exceeded 10% of its maximal velocity for a consecutive duration of 100 ms, and then tracing back the point in time in which the difference between velocity samples was positive (positive acceleration). Movement offset was set at the point in time after the maximal grip aperture velocity and before the point in time of the presentation of the second tone, in which the grip aperture’s tangential velocity was lower than 10% of its maximal velocity for a consecutive duration of 100 ms, and then by tracing forward the time in which the difference between velocity samples was negative (negative acceleration). The determination of the aperture and the points of onset and offset was visually supervised. Each aperture trace was animated using a stick diagram, and the onset and offset markers were presented with respect to the grip aperture velocity and could be manually adjusted by the operator.

To analyze grip apertures, each movement was divided into 10 equal intervals (10–100%). The average grip aperture and JNDs were calculated for each interval and for each object size separately. As in previous studies, JNDs were measured by the within-subject standard deviation of the aperture (Ganel et al., 2008). The adherence to Weber’s law was measured with a within-subject planned comparison test of the linear component of object size during each percentile of the movement, with emphasis on the more crucial, second part of the normalized movement (60–100%). JNDs were also computed at the point in time in which MGA was achieved. The analysis of the JND data at different time points within the movement was conducted to account for potential issues related to time-dependent scaling of the MGA during virtual grasping. Specifically, while the MGA is considered a reliable measure of the sensitivity of aperture to physical objects size in 3D grasping, accumulating evidence suggests that the MGA is a less reliable measure of performance during 2D grasping (Afgin et al., 2017; Ozana et al., 2018; Ozana and Ganel, 2018, 2019b). Additional kinematic aspects of the movement were also calculated: Reaction time (RT), which reflects the time between the go signal and between movement initiation, the absolute time to MGA (tMGA), and the total movement time (MT). The possible effect of practice was tested by comparing JNDs and grip apertures across the three sequential experimental blocks. The main independent variables were therefore, block (block 1, block 2, block 3), normalized movement time (10 levels), and object size (15, 25, 35, 45 cm).

We applied a correction for outliers on each participant’s aperture data. Trials in which the grip apertures at the point of movement completion were 2.5 standard deviations higher or lower than the participant’s mean aperture for the same object were removed from the analysis. The correction resulted in the exclusion of less than 2% of the trials.

### Results

#### Movement Profile

**FIGURE 2:**
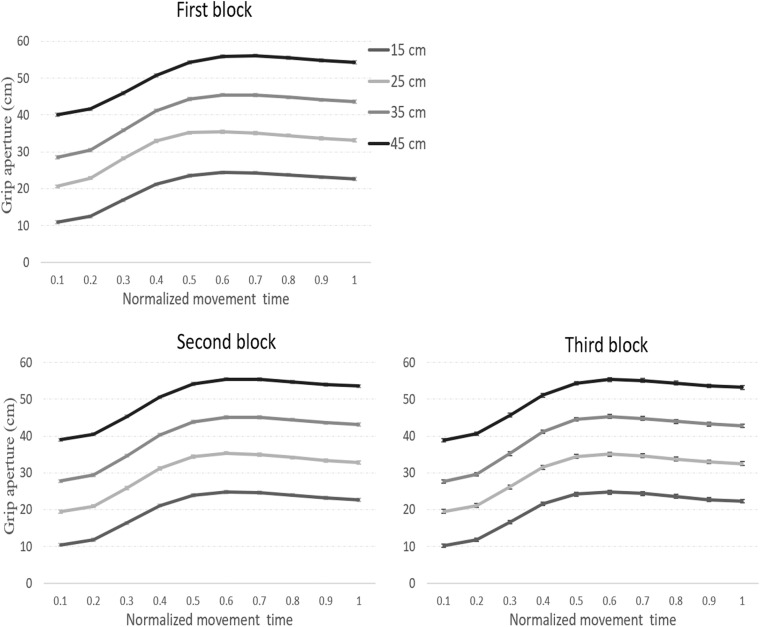
Average grip apertures across the three sequential experimental blocks in Experiment 1. Throughout the three blocks, apertures reflected the size differences between the objects. Error bars represent confidence intervals in repeated measures ANOVAs (Jarmasz and Hollands, 2009).

Average grip apertures (for each interval) toward the virtual targets are presented in Figure 2. As can be seen in the figure, grip apertures reflected the size differences between the objects. First, a repeated-measures ANOVA with block (3 levels), normalized movement time (10 levels), and object size (15, 25, 35, 45 cm) as within-subject independent variables was conducted on the *grip aperture data.* A Greenhouse-Geisser correction was applied for cases in which sphericity assumption was violated (based on Mauchly’s Test of Sphericity). The main effect of block was not significant [*F*_(1.1,17.3)_ = 1.3, *p* = 0.28], indicating that grip apertures were stable across blocks. Significant main effects of normalized movement time [*F*_(1,7,22.7)_ = 160.5, *p* < 0.001, η*_*p*_*^2^ = 0.92] and object size [*F*_(1.6,21.2)_ = 4,719, *p* < 0.001, η*_*p*_*^2^ = 0.99] showed that grip apertures changed throughout the movement and corresponded to the target size. The interaction between block and movement was not significant [*F*_(18,234)_ = 1.3, *p* = 0.18]. Yet, a significant interaction between block and size [*F*_(6,78)_ = 2.4, *p* = 0.03, η*_*p*_*^2^ = 0.15], indicated that grip apertures differently corresponded to size at different blocks of the experiment. There was a significant interaction between movement and size [*F*_(27,351)_ = 11.4, *p* < 0.001, η*_*p*_*^2^ = 0.47], indicating that sensitivity to size developed throughout the movement. A significant three-way interaction [*F*_(54,702)_ = 1.6, *p* < 0.01, η*_*p*_*^2^ = 0.11], showed that the time-dependent scaling of the aperture differed across blocks. MGAs also showed sensitivity to the target’s size [*F*_(1.3,17.1)_ = 4,536, *p* < 0.001, η*_*p*_*^2^ = 0.99] (26, 37, 47, 57 cm from the smallest to the largest object, respectively).

The response time data for the three blocks is presented in Table 1. A repeated-measures ANOVA test with block and size as the independent variables was conducted on RT data. The main effect of block was not significant [*F*_(1.1,15.4)_ =1.8, *p* = 0.17]. A significant effect of object size [*F*_(3,39)_ = 9.8, *p*< 0.001, η*_*p*_*^2^ = 0.43], indicated that time to initiate the movement differed between the different target sizes (461, 444, 460, 436 ms). There was a significant interaction, however, between block and size, that showed that the relation between RT and size differed across the three blocks [*F*_(679)_ = 3.8, *p* < 0.01, η*_*p*_*^2^ = 0.22]. Unlike the RT data, a repeated-measures ANOVA on tMGAs showed a significant effect of block [*F*_(1.4,18.5)_ = 5.3, *p* = 0.01, η*_*p*_*^2^ = 0.30] (908, 859, 836 ms, for the first, second, and third block, respectively), which indicates that time to reach maximum grip aperture differed in different blocks. The main effect of size was not significant [*F*_(1.3,17)_ = 2.2, *p* = 0.99]. Yet, a significant interaction between block and size [*F*_(3,39.8)_ = 4.9, *p*< 0.001, η*_*p*_*^2^ = 0.27] showed that the relation between the tMGA and size was different across blocks. Finally, a similar analysis of the MT data showed a significant main effect of block [*F*_(1.2,15.6)_ = 12, *p <* 0.001, η*_*p*_*^2^ = 0.48]. *Post hoc* test with Bonferroni correction showed that mean difference between the first block (1,784 ms) and the second block (1,640 ms) [*t*_(11)_ = 4.8, *p <* 0.001], and between the first block and the third block (1,604 ms) [*t*_(11)_ = 3.4, *p* = 0.01] were both significant. These results indicate that the time to complete the movement was relatively slower in the first block. A test of the within-subject contrasts showed a significant linear trend, indicating that the time to complete the movement decreased with practice [*F*_(1,13)_ = 11, *p* < 0.001, η*_*p*_*^2^ = 0.47] (1,784, 1,640, 1,601 ms, respectively). The main effect of size did not reach statistical significance [*F*_(3,39)_ = 2.6, *p* = 0.06]. The interaction between block and size was also not significant [*F*_(2.8,37.2)_ = 0.90, *p* = 0.48]. We also note that times to complete the movement (MTs) were considerably longer and that maximum grip apertures arrived at a relatively early stage in the normalized movement (52%) compared to previous 3D grasping (Jakobson and Goodale, 1991; Smeets and Brenner, 1999; see Ozana et al., 2018, for similar findings).

**TABLE 1:** Mean RTs, tMGAs, and MTs in ms (± 1 SD) for each of the objects in Experiment 1.

		15 cm	25 cm	35 cm	45 cm
Block 1	RT	468 ± 72	468 ± 84	494 ± 109	449 ± 84
	tMGA	957 ± 101	840 ± 98	905 ± 104	957 ± 101
	MT	1,777 ± 224	1,797 ± 260	1,759 ± 165	1,803 ± 194
Block 2	RT	456 ± 73	437 ± 76	431 ± 65	415 ± 62
	tMGA	839 ± 95	842 ± 72	883 ± 83	869 ± 110
	MT	1,589 ± 116	1,662 ± 114	1,651 ± 126	1,658 ± 193
Block 3	RT	460 ± 106	426 ± 77	456 ± 115	445 ± 122
	tMGA	841 ± 144	828 ± 107	822 ± 118	853 ± 131
	MT	1,584 ± 115	1,620 ± 82	1,600 ± 130	1,611 ± 114

Thus, while the movement showed some characteristics that were similar to normal 3D grasping control and while apertures were sensitive to the target’s size, other kinematics of the movement were somewhat atypical. There were also differences among several kinematic aspects across different blocks of the movement, which suggest that practice with the task had an effect on grasping performance. The effect of practice on JNDs will be further explored in the next section.

#### JNDs

**FIGURE 3:**
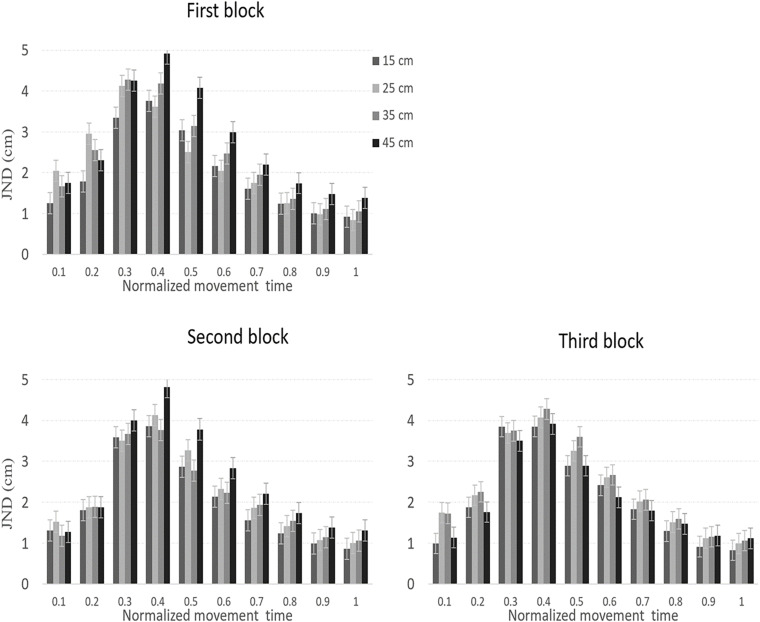
JNDs across the three sequential experimental blocks in Experiment 1. In all blocks, JNDs increased with size throughout most of the movement, in line with Weber’s law. Error bars represent confidence intervals in repeated measures ANOVAs (Jarmasz and Hollands, 2009).

JNDs across the movement are presented in Figure 3. JNDs during the second part of the movement increased with the target size, in line with Weber’s law.

As we mentioned earlier, the main analysis of the JNDs data was conducted on the second part of the normalized movement time. A repeated-measures ANOVA with block, normalized movement time (5 levels, 60–100%), and object size as independent variables was conducted on the data. The main effect of block [*F*_(1.4,18.5)_ = 0.3, *p* = 0.97] was not significant, which indicates that practice did not affect the overall size of the JND. There were significant main effects of normalized movement time [*F*_(1,14.2)_ = 56, *p*< 0.001, η*_*p*_*^2^ = 0.81] and object size [*F*_(3,39)_ = 11.1, *p* < 0.001, η*_*p*_*^2^ = 0.46], which indicate that JNDs differed across the normalized movement, and for different object sizes. The interaction between block and size [*F*_(3.5,45.9)_ = 2.2, *p* = 0.08], however, was not significant. The interaction between block and movement [*F*_(2.7,35.8)_ = 0.1, *p* = 0.99], between movement and size [*F*_(3.7,48.5)_ = 0.5, *p* = 0.85] and the three-way interaction were also non-significant [*F*_(24,312)_ = 0.4, *p* = 0.98]. Lastly, the main effect of object size at the point in which MGAs were achieved was not significant [*F*_(3,39)_ = 1.1, *p* = 0.34].

To test for adherence to Weber’s law, we performed a within-subject planned comparison test of the linear component of object size during the second part of the movement. The test showed that JNDs linearly increased with size [*F*_(1,13)_ = 33, *p* < 0.001, η*_*p*_*^2^ = 0.72] (1.4, 1.5, 1.6, 1.8 cm) in line with Weber’s law. As discussed earlier, the (linear) pattern of the JNDs could be confounded by the velocity of the grip aperture (Ganel et al., 2014; Ozana and Ganel, 2019b). Therefore, a similar repeated-measures ANOVA test with block, normalized movement time, and size as independent variables was conducted on the aperture velocities during the second part of the normalized movement. Importantly, the main effect of block was not significant [*F*_(1.1,14.9)_ = 2.2, *p* = 0.12]. The main effects of size [*F*(3, 39) = 2.7, *p* = 0.058] and the interaction between block and size [*F*_(2.7,35.6)_ = 2.2, *p* = 0.09] approached significance. The reader should note that these trends toward significance might have resulted from performance during the first block (see Table 2). Indeed, as can be seen in the table, in the first block (but not the second or third blocks), aperture velocity linearly increased with size in each percentile of the second part of the movement. Also note that the linear pattern of JNDs is still maintained when excluding the first block from the analysis [*F*_(1,13)_ = 11, *p* = 0.01, η*_*p*_*^2^ = 0.46]. A potential speed-precision tradeoff effect between aperture velocities and JNDs could therefore be ruled out from the final two blocks but could have affected performance in the first block (Table 2). Finally, to provide a thorough investigation of the pattern of adherence to Weber’s law across different stages of the normalized movement, planned comparisons of the linear component of size were conducted separately on the JNDs and aperture velocities data for each movement percentile and in each block. The results are also shown in Table 2. Note, that JNDs increased with the target size at the final stages of the movement.

**TABLE 2:** Planned comparisons of the linear component of size for the JNDs and aperture velocity data in each of the normalized movement percentiles in Experiment 1.

Experiment 1 (No haptic feedback)												
Block 1					Block 2				Block 3			
	JND		Aperture velocity		JND		Aperture velocity		JND		Aperture velocity	
	*F*	η*_*p*_*^2^	*F*	η*_*p*_*^2^	*F*	η*_*p*_*^2^	F	η*_*p*_*^2^	*F*	η*_*p*_*^2^	*F*	η*_*p*_*^2^
10%	0.8	0.06	6.3*	0.32	0.3	0.02	0.3	0.02	1.1	0.11	0.8	0.06
20%	1.7	0.11	0.8	0.007	0.4	0.03	1.3	0.09	0.6	0.005	0.5	0.04
30%	6.2**	0.32	5.8*	0.31	3.4	0.20	1.3	0.09	0.4	0.03	1.5	0.10
40%	9.8**	0.43	7.8*	0.37	5.1*	0.28	3.3	0.20	0.6	0.04	2.9	0.18
50%	7.4**	0.36	6.4*	0.33	5.1*	0.28	7.5*	0.36	0.4	0.03	2.6	0.17
60%	8.6**	0.40	7.3*	0.36	3.7	0.22	1.9	0.12	0.5	0.004	0.3	0.003
70%	16.9**	0.56	5.7*	0.30	21.1**	0.62	1.2	0.08	0.1	0.009	0.3	0.003
80%	12.5**	0.49	4.2	0.24	28.6**	0.68	0.7	0.05	2.2	0.15	0.05	0.000
90%	15.3**	0.54	5.6*	0.30	27.2**	0.67	0.4	0.03	5.2*	0.28	0.2	0.02
100%	13.2**	0.50	13.1**	0.50	23.9**	0.64	0.8	0.06	7.9**	0.38	0.8	0.06

**^∗^p < 0.05, ^∗∗^P < 0.01.**

The results of Experiment 1 show that when tactile feedback is not available at the end of the movement, actions in VR are subjected to perceptual regularities of object size, and obey to Weber’s law. This pattern of results indicates that grasping movements relied on less effective, relative computations of the target, compared to normal 3D grasping. The results also show several variations in kinematic aspects of the movement between blocks. Yet, we note that these differences between the blocks along the pattern of adherence to Weber’s law did not reach statistical significance. Experiment 2 was designed to examine the role of haptic feedback in the adherence to Weber’s law. The experimental design was similar to the one used in Experiment 1, but now object-specific haptic information was provided upon touch.

## Experiment 2

### Materials and Methods

#### Participants

Fourteen additional participants (six males, average age = 25.2, *SD* = 1.3) participated in the experiment for the same monetary compensation as in Experiment 1. The result of one participant was excluded from the analysis because she failed to follow the experimental instructions.

#### Procedure and Design

The procedure was similar to the one used in Experiment 1, except that now a matching size set of 3D objects (15, 25, 35, 45 cm) made out of polyester, were embedded in the virtual environment in a location that matched that of the virtual target. In each trial, one 3D object was placed by the experimenter prior to movement initiation and provided object-specific haptic information upon touching the virtual target. Less than 2% of the trials were considered as outliers based on the same criterion used in Experiment 1.

### Results

#### Movement Profile

**FIGURE 4:**
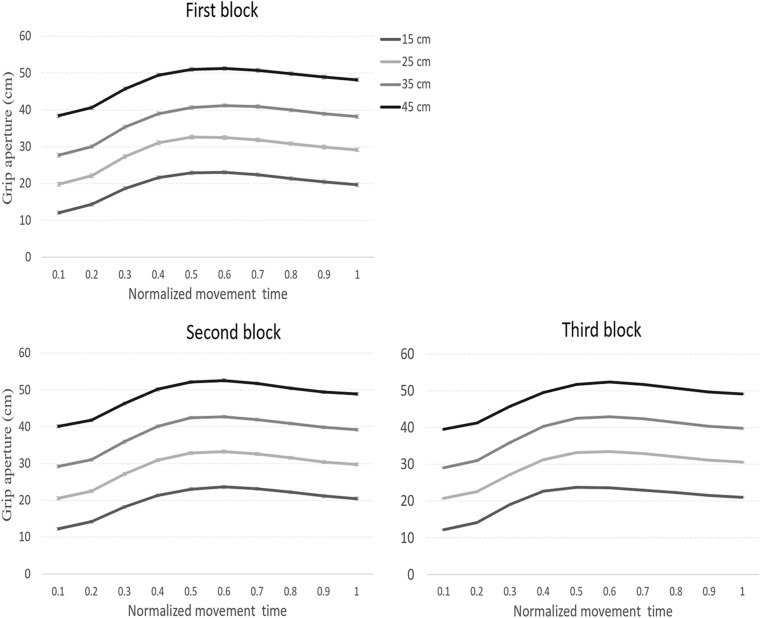
Average grip apertures across the three sequential experimental blocks Experiment 2. Grip apertures reflected the size differences between the objects. Error bars represent confidence intervals in repeated measures ANOVAs (Jarmasz and Hollands, 2009).

Average grip apertures in Experiment 2 are presented in Figure 4. As in Experiment 1, grip apertures reflected the size differences between the objects. A repeated-measures ANOVA with size, block, and normalized movement time (10 levels) was conducted on the grip aperture data. The main effect for block was not significant [*F*_(1.3,17.3)_ = 1.3, *p* = 0.28]. There were significant main effects of normalized movement time [*F*_(1.7,22.7)_ = 160.5, *p* < 0.001, η*_*p*_*^2^ = 0.92], and of object size [*F*_(1.6,21.2)_ = 4719, *p <* 0.001, η*_*p*_*^2^ = 0.99]. The interaction between block and movement was not significant [*F*_(18,234)_ = 1.3, *p* = 0.18]. Yet, as in Experiment 1, there was a significant interaction between block and size [*F*_(6,78)_ = 2.4, *p =* 0.03, η*_*p*_*^2^ = 0.15], which indicates that grip apertures were shaped differently to different target sizes at different blocks of the movement. The interaction between movement and size was significant [*F*_(27,351)_ = 11.9, *p* < 0.001, η*_*p*_*^2^ = 0.47]. There was also a significant three-way interaction [*F*_(54,702)_ = 1.6, *p <* 0.01, η*_*p*_*^2^ = 0.11], which indicates that the relation between the movement and grip aperture with respect to size differed across the blocks. An analysis of the MGA data showed a significant main effect for size [*F*_(1.4,17.3)_ = 2469, *p* < 0.001, η*_*p*_*^2^ = 0.99] (25, 35, 44, 54 cm, from the smallest to the largest target, respectively).

Response times for each block in Experiment 2 are presented in Table 3. A repeated-measures ANOVA of the RT data revealed a significant main effect of block [*F*_(2,24)_ = 6.6, *p <* 0.01, η*_*p*_*^2^ = 0.35]. *Post hoc* tests with Bonferroni correction showed that mean difference between the first block (450 ms) and the second block (424 ms) did not reach statistical significance [*t*_(10)_ = 2, *p* = 0.18]. However, the mean difference between the first and third block (409 ms) was significant [*t*_(10)_ = 3.1, *p* = 0.02]. There was also a significant main effect of size [*F*_(2,24)_ = 6.8, *p* < 0.01, η*_*p*_*^2^ = 0.36]. The *post hoc* tests with Bonferroni correction did not show a significant difference between the mean score of the 15 cm target (smallest target, 448 ms) and the 25 cm target (418 ms) [*t*_(10)_ = 2.8, *p* = 0.08], or between the smallest target and the 45 cm target (425 ms) [*t*_(10)_ = 2.5, *p* = 0.15]. However, there was a significant difference between the mean score of the smallest target and that of the 35 cm target (421 ms) [*t*_(10)_ = 3.2, *p* = 0.04]. The interaction between block and size [*F*_(2,24)_ = 1.2, *p* =0.27] was not significant. Analysis of the tMGA data also showed significant main effects of block [*F*_(2.24)_ = 13.4, *p <* 0.001, η*_*p*_*^2^ = 0.52] (779, 729, 697 ms, respectively) and of size [*F*_(3,36)_ = 5.1, *p* < 0.01, η*_*p*_*^2^ = 0.30] (721, 723, 763, 731 ms, respectively), which indicate that the time in which the MGA was achieved was different in the different blocks and for the different sizes. The interaction between block and size was also significant [*F*_(2,24.6)_ = 4.5, *p* = 0.02, η*_*p*_*^2^ = 0.27], indicating that the relation between tMGA and size differed between the blocks. Unlike RTs and tMGAs, an analysis of the MT data did not show a significant main effect of block [*F*_(2,24)_ = 2.7, *p* = 0.08] or of size [*F*_(2.1,25.8)_ = 1, *p* = 0.36]. A significant interaction [*F*_(2.27)_ = 3.9, *p* = 0.02, η*_*p*_*^2^ = 0.24], however, indicated that time to complete the movement in relation to target size differed between the blocks.

**TABLE 3:** Mean RTs, tMGAs, and MTs (±1 SD) in ms for each of the objects in Experiment 2.

		15 cm	25 cm	35 cm	45 cm
Block 1	RT	483 ± 76	435 ± 52	436 ± 50	446 ± 32
	tMGA	741 ± 92	781 ± 144	842 ± 132	753 ± 87
	MT	1,575 ± 380	1,624 ± 307	1,654 ± 277	1,591 ± 332
Block 2	RT	433 ± 60	415 ± 38	421 ± 22	431 ± 59
	tMGA	742 ± 72	703 ± 55	748 ± 108	714 ± 79
	MT	1,594 ± 299	1,496 ± 219	1,543 ± 279	1,491 ± 223
Block 3	RT	428 ± 39	404 ± 31	406 ± 30	399 ± 31
	tMGA	680 ± 83^‘^	685 ± 67	699 ± 73	725 ± 92
	MT	1,505 ± 253	1,461 ± 199	1,470 ± 214	1,507 ± 227

The overall differences along the response time pattern in experiments 1 and 2 did not reach statistical significance. A mixed-model ANOVA of MTs with block and size as independent factors did not reveal a significant main effect of experiment [*F*_(1,25)_ =3.4, *p* = 0.07]. Similarly, tests conducted on the relative time of the MGA in the normalized movement [*F*_(1,25)_ = 3.4, *p* = 0.07], and on RTs [*F*_(1,25)_ = 2.9, *p* = 0.09], did not show a significant effect of experiment. We note that as in Experiment 1, the time to complete the movement toward the virtual target was relatively longer than in typical 3D grasping tasks, and that MGAs arrived at a relatively early part of the movement (48%) (Jakobson and Goodale, 1991; Smeets and Brenner, 1999).

#### JNDs

**FIGURE 5:**
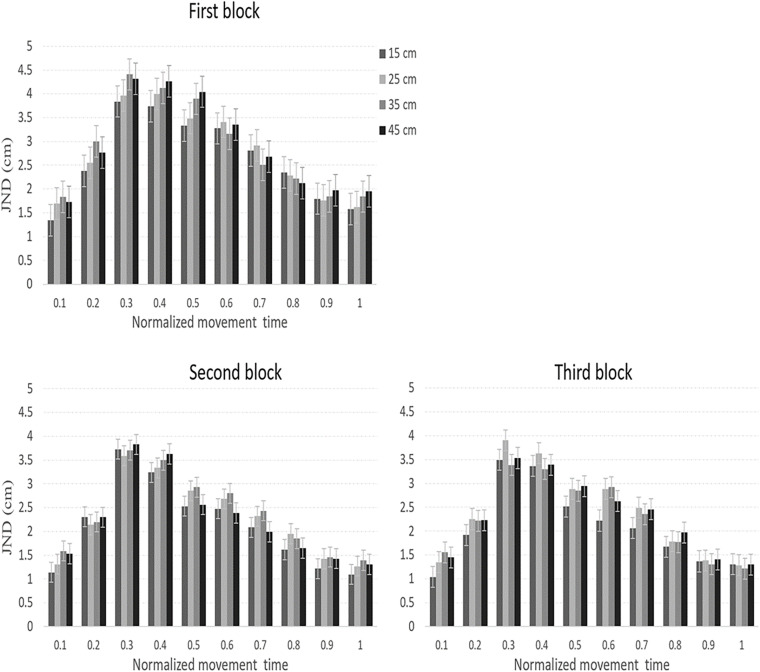
JNDs across the three sequential experimental blocks in Experiment 2. In all blocks, and unlike the results of Experiment 1, JNDs generally did not increase with size, in violation of Weber’s law. Error bars represent confidence intervals in repeated measures ANOVAs (Jarmasz and Hollands, 2009).

JNDs across the normalized movement trajectory are presented in Figure 5. As can be seen in the figure, and unlike the pattern of results in Experiment 1, JNDs did not increase with size at the final stages of the movement, in violation of Weber’s law.

A repeated-measures ANOVA with block, normalized movement time during the second part of the movement (5 levels), and object size as independent variables was conducted on the JND data. There was a significant main effect for block [*F*_(1.3,13.1)_ = 5.5, *p* = 0.03, η*_*p*_*^2^ = 0.31]. Simple within-subject contrasts revealed that JNDs in the first block were significantly larger than the second block [*F*_(1,12)_ = 6.9, *p* = 0.02, η*_*p*_*^2^ = 0.36] and the third block [*F*_(1,12)_ = 4.7, *p* = 0.05, η*_*p*_*^2^ = 0.28] (2.5, 1.8, 1.9 cm, for the first, second, and third block, respectively), indicating that practice decreased the overall size of JNDs (and therefore, increased precision in the task). There was a main effect of movement [*F*_(1.1,13.2)_ = 21.6, *p* < 0.001, η*_*p*_*^2^ = 0.64], which indicates that JNDs values were different at different stages of the movement. Yet, the main effect of size was not significant [*F*_(3,36)_ = 1.2, *p* = 0.31]. Importantly, the interactions between block and movement [*F*_(2.2,27.1)_ = 0.94, *p* = 0.48], block and size [*F*_(6,72)_ = 0.64, *p =* 0.69], and between movement and size [*F*_(3.3,40.6)_ = 1.7, *p* = 0.*16*], were not significant. A significant three-way interaction [*F*_(24,288)_ = 1.6, *p* = 0.03, η_*p*_^2^ = 0.11], indicated that the relation between movement and size was different at different blocks. The main effect of size at the point of MGA was not significant [*F*_(3,36)_ = 1, *p* = 0.38] (2.2, 2.4, 2.3, 2.2 cm, from the smallest to the largest object).

**FIGURE 6:**
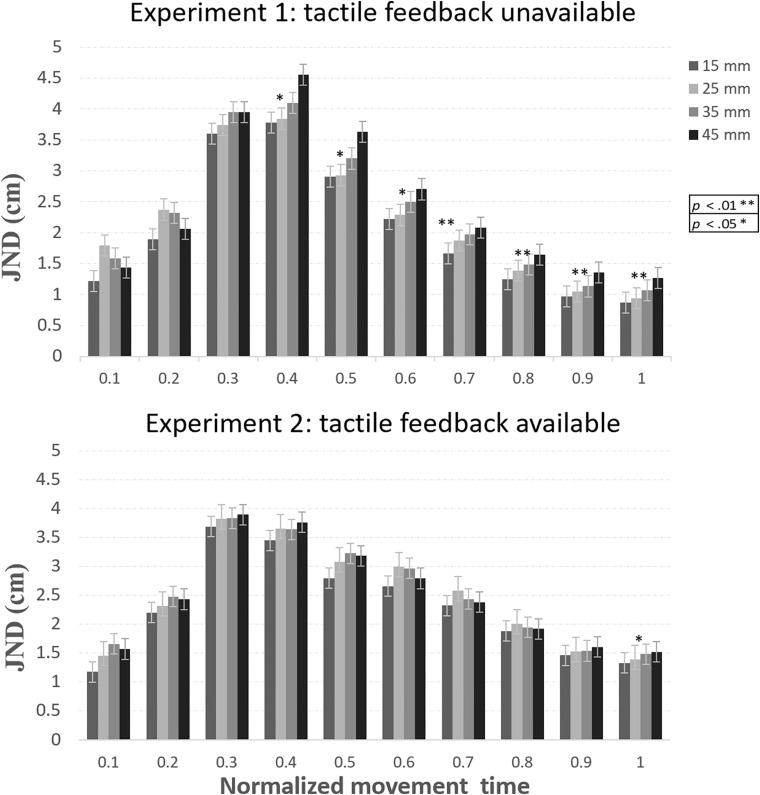
Average JNDs across the three blocks in Experiments 1 and 2. In Experiment 1, JNDs adhered to Weber’s law throughout most stages of the movement. Conversely, in Experiment 2, Weber’s law was violated in most stages of the movement. Error bars represent confidence intervals in repeated measures ANOVAs (Jarmasz and Hollands, 2009). *p < 0.05; **p < 0.01.

Unlike the results of Experiment 1, a within-subject planned comparison test of the linear component of size did not show a linear increase in JNDs with size [*F*_(1,12)_ = 1.1, *p* = 0.30], in violation of Weber’s law. To test if the JND pattern in the current experiment was significantly different from that obtained in Experiment 1 (when no feedback was allowed), a mixed ANOVA with experiment as a between-subjects factor, and block, normalized movement time, and size as a within-subject factors was conducted on the JND data. Notably, significant interaction between experiment and size [*F*_(2.8,71.4)_ = 3.4, *p* = 0.02, η*_*p*_*^2^ = 0.12] indicated that the JND pattern was different between the two experiments (see Figure 6). Planned comparisons of the linear component of size for each percentile of the movement are presented in Table 4. Note that as in Experiment 1, the pattern of scalar variability of JNDs with size that was obtained in block 1 was confounded by the velocity of the grip aperture. Therefore, it is unclear whether the linear increase of the JNDs in the first block reflects genuine adherence to Weber’s law. We also note that there was a peculiar pattern of an increase of JNDs with size at 10% of the movement. However, this unexpected pattern probably does not represent genuine adherence to Weber’s law because JNDs did not show stable increase with size throughout the entire size range (see Figures 5, 6).

**TABLE 4:** Planned comparisons of the linear component of size for the JNDs and aperture velocity data in each of the normalized movement percentiles in Experiment 2.

Experiment 2 (haptic feedback)												
Block 1					Block 2				Block 3			
	JND		Aperture velocity		JND		Aperture velocity		JND		Aperture velocity	
	*F*	η*_*p*_*^2^	*F*	η*_*p*_*^2^	*F*	η*_*p*_*^2^	*F*	η*_*p*_*^2^	*F*	η*_*p*_*^2^	*F*	η*_*p*_*^2^
10%	2.3	0.16	0.2	0.09	7.9**	0.40	0.00	0.02	6.5*	0.35	1.0	0.07
20%	2.4	0.16	3.2	0.21	0.01	0.00	0.1	0.01	0.6	0.005	0.04	0.004
30%	1.6	0.12	6.9*	0.36	0.1	0.01	4.6	0.27	1.0	0.08	2.3	0.16
40%	1.3	0.10	2.2	0.16	0.9	0.07	1.1	0.08	0.5	0.004	6.1*	0.33
50%	2.9	0.20	0.01	0.002	0.02	0.002	1.0	0.08	1.4	0.10	2.2	0.15
60%	0.0	0.00	2.9	0.19	0.7	0.006	1.9	0.12	1.4	0.10	3.3	0.21
70%	0.5	0.04	0.1	0.01	0.9	0.007	4.9*	0.29	1.5	0.11	7.9*	0.39
80%	0.6	0.05	2.2	0.15	0.0	0.00	2.8	0.19	3.0	0.20	4.6	0.28
90%	0.8	0.06	3.5	0.22	1.2	0.09	3.0	0.20	0.1	0.001	5.8*	0.32
100%	5.6*	0.32	8.5*	0.41	1.7	0.12	0.9	0.07	0.7	0.006	3.4	0.22

*^∗^*P* < 0.05, ^∗∗^*P* < 0.01.*

The results of Experiment 2 suggest that the availability of accurate haptic information upon touch contributes to efficient performance in VR in terms of resistance to perceptual regularities. Unlike Experiment 1 (and similarly to 3D grasping), interactions that entail obtaining accurate haptic feedback from the target were refractory to Weber’s law, which could indicate more analytic computation of size.

The results also suggest that practice affected participants’ performance in the task. JNDs in the first block were significantly larger than the JNDs in the remaining blocks, which is an indication of poorer visual resolution to size (but could also indicate more stochastic noise). It is unclear, to which extent did training contributed to the overall pattern of resistance to Weber’s law. Indeed, while during the first block (but not for the remaining blocks) JNDs at the point of movement completion adhered to Weber’s law, a similar pattern of aperture velocity also emerged at this point in time. As we mentioned earlier, such co-occurrence could serve as an alternative account to a pattern of scalar variability; it may simply reflect a speed-precision tradeoff effect (Foster and Franz, 2013; Ganel et al., 2014; Ozana and Ganel, 2019b). The possible effect of training on the adherence to Weber’s law in VR haptic systems should be further explored in future studies.

Finally, it should be noted that while actions in the feedback condition showed a typical pattern of resistance to Weber’s law, they were still some divergences along several kinematic aspects of the movement. For instance, just as in Experiment 1, movements were relatively slow compared to actions directed to 3D objects. These differences might be attributed to participants’ unfamiliarity with the computerized environment, which could be further attenuated via extensive training.

## General Discussion

In the current study, we examined the nature of visuomotor interactions with digitized objects in a virtual environment. The results suggest that haptic information affects the way visual information is processed within virtual settings. When haptic information was not available, grip apertures showed an abnormal pattern and were subjected to a perceptual heuristic of relative size, obeying Weber’s law. However, when grasping movements were accompanied with accurate haptic feedback upon touch, Weber’s law was violated throughout most stages of the movement, a pattern that also characterizes normal 3D grasping.

The idea that the visual system is divided into two functionally distinct pathways has gained ample support from neurological and behavioral studies (for a review, see Milner and Goodale, 2008). For example, previous studies showed that Weber’s law does not fully apply to bimanual and precision grasping movements, suggesting that the visual control of action relies on analytical coding of object size (Ganel et al., 2008, 2017; but see Smeets and Brenner, 2008 for an alternative account). However, recent studies have shown that when 2D objects are used as targets, actions are no longer immune to Weber’s law (Holmes and Heath, 2013; Ozana and Ganel, 2019a, b), as well as to other perceptual regularities (Chen et al., 2015; Freud and Ganel, 2015; Ozana and Ganel, 2018). These results suggest that the dissociation between action and perception does not extend to visuomotor interactions with virtual, 2D objects. The current results, however, show that virtual interactions in state-of-the-art virtual settings could still evade Weber’s law, provided that accurate haptic feedback is available upon touch. These findings indicate that crucial aspects of normal visuomotor control could generalize to interactions with virtual objects, given that such interactions provide a reliable sense of control. In consideration of previous studies, this suggests that the efficiency of the action toward virtual targets in terms of resistance to task-irrelevant information depends on the degree of authenticity provided by the virtual system (Afgin et al., 2017; Ozana et al., 2018). Indeed, it could be argued that interactions within a 3D environment that entails immediate haptic feedback from a virtual object can be considered as more reliable than other types of interactions with virtual objects, interactions that are performed on touch screens or in remote virtual settings and do not provide authentic haptic feedback. This idea is also in line with the suggestion that visuomotor computations are influenced by the potential outcome of the interaction (Hosang et al., 2016; Freud et al., 2018; Ozana and Ganel, 2019a).

It should be noted, however, that while the results of the current study suggest that actions toward virtual targets could be performed in an analytical and efficient manner, the extent to which this could apply to present immersive technologies remains unclear. For example, in an attempt to maximize the potential effect of tactile feedback from the virtual target on visuomotor control, haptic information in our feedback condition was provided from physical objects of matched sizes, which were embedded in the virtual environment. Furthermore, we used experimental instructions that encouraged participants to grasp the virtual targets the same way they grasp real 3D objects. It is unclear whether current tactile virtual technology (e.g., feedback from tactile gloves) can evoke a similar sense of interaction. Such virtual feedback devices, for example, may still lack in terms of precision and timing delays, which could compromise the sense of agency, leading to inefficient performance (Rohde and Ernst, 2016), and the usage of relative metrics (Afgin et al., 2017). Virtual interactions may also entail different gestures other than grasping, which could rely on different computations of size (Ozana et al., 2020). Hence, further research should explore the mechanisms that permit normal performances within VR.

Previous studies highlighted the role of tactile feedback in 2D and 3D grasping (Bingham et al., 2007; Johansson and Flanagan, 2009; Whitwell et al., 2014, 2016; Davarpanah Jazi et al., 2015; Hosang et al., 2016; Cesanek and Domini, 2017; Kopiske et al., 2017). For instance, it was argued that the provision of terminal haptic feedback could support analytic visuomotor control via visuo-haptic calibration on subsequent trials (Davarpanah Jazi et al., 2015; Hosang et al., 2016). In 2D grasping, initial support for this idea was obtained in a study that utilized a delayed haptic-feedback design, in which a 3D object of matching size was placed between the participant’s index and thumb following movement completion. In line with the current results, the findings showed that actions in this delayed feedback condition violated Weber’s law (Hosang et al., 2016). However, the ecological validity of Hosang et al. (2016) results could be limited by the fact that haptic feedback was provided only after movements were completed. It could be argued that such atypical settings might encourage participants to treat the task of 2D grasping and 3D grasping as separate events. Indeed, as discussed in the introduction, these findings were inconsistent with the results of a more recent study from our lab in which immediate haptic feedback was provided during 2D grasping by haptic feedback simulator. In this study, actions obeyed Weber’s law throughout the entire movement trajectory, regardless of the availability of haptic information (Ozana et al., 2018). In a complementary manner, in a different study from our lab, participants performed grasping gestures toward different-sized 3D objects placed beyond a transparent glass. While the tactile feedback provided in this task was partial (similar to the feedback typically available in interactions with 2D objects), the results showed that grasping violated Weber’s law (Ozana and Ganel, 2019a). Thus, it seems that while tactile information can have an important role in grasping performance, efficient, analytic visuomotor control is not contingent only upon this source of information. Therefore, analytic visuomotor control probably depends on the availability of cross-modal, converging sources of information that are available in 3D grasping. Such visual and tactile cues may evoke a dedicated set of computations that support efficient motor control.

The potential effect of tactile feedback on grasping was also illustrated in recent studies conducted on DF, a patient who suffers from visual form agnosia due to bilateral damages to her ventral stream. Remarkably, although DF is unable to discriminate between different-sized objects she can accurately scale her fingers to grasp them, arguably relaying on her intact dorsal stream (see Whitwell et al., 2014). Importantly, however and in line with the results of Experiment 2, recent work suggests that DF’s (normal) performance in grasping tasks depends on her ability to obtain tactile information when grasping the target’s edges (Schenk, 2012). For example, when DF is asked to perform pantomime movements toward objects her fingers do not longer scale to the size of the target. This finding is in line with the current results, further supporting the idea that tactile feedback has a role in action-perception dissociations. We note, however, that DF’s visual processing might still differ substantially from that of healthy controls. For example, while the actions of healthy subjects are compromised when 2D objects (that only provide general tactile feedback from touching the flat surface) are used as targets, DFs show sensitivity to 2D and 3D targets during grasp (Whitwell et al., 2014).

An alternative explanation of the findings of the current study could be that bimanual grasping in rich VR environments relies on a double pointing (Smeets and Brenner, 2008). According to this account, grasping depends on independently pointing each digit to a different location, rather than encoding the object size. Indeed, this model could potentially explain why perceptual regularities about object size and context typically do not affect visuomotor control (Smeets et al., 2019). We note, however, that while the current results are consistent with this simple, double-pointing account, this proposal is not in line with previous evidence about 2D grasping, a task, which arguably should also involve directing the digits at two discrete locations. However, simple interactions with 2D objects (as well as with 3D virtual objects in Experiment 1) typically obey perceptual regularities (Holmes and Heath, 2013; Freud and Ganel, 2015; Ozana and Ganel, 2018, 2019a), which goes against a simple double pointing strategy account (but see, Smeets et al., 2019).

As in recent studies that involve virtual interactions with 2D objects, MGA data from our no-feedback condition did not reflect the pattern of adherence to Weber’s law obtained in the second part of the movement trajectory (Afgin et al., 2017; Ozana et al., 2018; Ozana and Ganel, 2019b). A possible reason for the inconsistency between the pattern of JNDs during MGAs and between the pattern of JNDs at the rest of the movement in 2D (but not in 3D) interactions, may be related to task requirements. In particular, in 3D grasping, MGAs are considered as basic and stable kinematic signature of grip apertures that reflects the safety margin required to firmly grasp the target object prior to lifting it up. Yet, actions that do not entail the grasping of physical objects do not require such safety margins. As a result, these interactions usually have a different movement profile, which lacks a reliable point in which MGAs are achieved (Ozana et al., 2018). To account for this issue, JNDs in the current study were measured at different intervals of the movement trajectory. The results showed that MGAs did not represent the pattern of JNDs in other stages of the movements, including the critical stage in which the fingers approached the target object. Therefore, together with previous findings, the current results suggest that when grasping is less typical, MGA may not provide a reliable measure of performance (Afgin et al., 2017; Ozana et al., 2018).

Another potential pitfall is related to the possible effect of the aperture velocity on the pattern of adherence to Weber’s law during virtual grasping. In a typical grasping task, participants are required to pinch their fingers together prior to movement initiation, a design that might encourage them to open their fingers faster to big compared to small objects. This relation between aperture velocity and size can lead to speed-precision tradeoff effects. Such effects may also lead to a decrease in precision (larger SDs) for bigger objects (Foster and Franz, 2013; Ganel et al., 2014; but see, Heath et al., 2012). In 3D grasping, this potential confound has been shown to affect early stages of the movement. However, in a recent study, we found that actions directed to 2D targets could be subjected to speed-precision tradeoff effects throughout the entire movement. Hence, in atypical grasping tasks, adherence to Weber’s law could reflect the relation between the aperture’s velocity and SD rather than the visual resolution of the response (Ozana and Ganel, 2019b).

To summarize, actions toward 3D and 2D targets typically show distinctive patterns of adherence to psychophysical principles. Actions toward 2D objects are typically subjected to perceptual regularities, the same regularities that do not affect normal 3D grasping. Here, we showed that this dissociation between action and perception extends to advanced immersive surroundings in which accurate haptic feedback is available upon touch. These results suggest that the inefficient control of action, found in various types of 2D visuomotor interactions, could be attributed to a reduced sense of interaction with the target, which might lead to atypical behavior. The presence of visual and haptic cues from the environment could facilitate an elevated sense of interaction, and enable more accurate and natural grasping performance in a virtual environment.

## Data Availability Statement

The raw data supporting the conclusions of this article will be made available by the authors, without undue reservation.

## Ethics Statement

The studies involving human participants were reviewed and approved by Institutional Ethics Committee, Ben-Gurion University of the Negev. The patients/participants provided their written informed consent to participate in this study.

## Author Contributions

TG, AO, and SB planned the experiments, analyzed the data, reviewed the manuscript, and contributed to edits and updates of the manuscript. AO ran the experiments, performed initial data analyses, and wrote the initial draft of the manuscript. All authors contributed to the article and approved the submitted version.

## Conflict of Interest

The authors declare that the research was conducted in the absence of any commercial or financial relationships that could be construed as a potential conflict of interest.
